# Rapid recovery of serratus anterior muscle function after microneurolysis of long thoracic nerve injury

**DOI:** 10.1186/1749-7221-2-4

**Published:** 2007-02-09

**Authors:** Rahul K Nath, Sonya E Melcher

**Affiliations:** 1 Texas Nerve and Paralysis Institute, Houston, Texas, USA

## Abstract

**Background:**

Injury to the long thoracic nerve is a common cause of winging scapula. When the serratus anterior muscle is unable to function, patients often lose the ability to raise their arm overhead on the affected side.

**Methods:**

Serratus anterior function was restored through decompression, neurolysis, and tetanic electrical stimulation of the long thoracic nerve. This included partial release of constricting middle scalene fibers and microneurolysis of epineurium and perineurium of the long thoracic nerve under magnification. Abduction angle was measured on the day before and the day following surgery.

**Results:**

In this retrospective study of 13 neurolysis procedures of the long thoracic nerve, abduction is improved by 10% or greater within one day of surgery. The average improvement was 59° (p < 0.00005). Patients had been suffering from winging scapula for 2 months to 12 years. The improvement in abduction is maintained at last follow-up, and winging is also reduced.

**Conclusion:**

In a notable number of cases, decompression and neurolysis of the long thoracic nerve leads to rapid improvements in winging scapula and the associated limitations on shoulder movement. The duration of the injury and the speed of improvement lead us to conclude that axonal channel defects can potentially exist that do not lead to Wallerian degeneration and yet cause a clear decrease in function.

## Background

Scapular winging due to injury of the long thoracic nerve (LTN) can have significant and debilitating effects on arm mobility. The serratus anterior muscle, innervated by the LTN, is responsible for stabilizing the scapula against the thoracic wall. Additionally, during abduction of the arm, the scapula is moved and stabilized by the serratus anterior to allow the humeral head to rotate. In studies of scapulothoracic motion, an increasing angle of humeral elevation correlates with increasing serratus anterior contraction[1,2]. Patients with injury of the LTN may be unable to abduct and flex the arm into upward rotation above 90° at the shoulder, and this is exacerbated when significant weight is added. This functional problem does not always resolve upon conservative treatment with physical therapy, and the literature is unclear on the role of therapy alone in recovering from this significant nerve injury.

The LTN is physically delicate and thin and transverses the middle scalene muscle in the neck where it is susceptible to stretching and compression[3,4]. A common cause of LTN injury is sudden lifting of a weight which is generally heavy, but may be as light as an infant child being carried by the mother. The mechanism seems to be compression of the LTN within the scalene muscle exacerbated by a stretch force along the course of the nerve [3-6]. Direct blunt force trauma to the upper torso and neck and heavy lifting can also injure the LTN and cause winging of the scapula. Other common causes are inadequate intraoperative positioning of a surgical patient and longstanding compression of the neck and shoulder areas while obtunded for more than several hours. Onset can sometimes be traced to a single traumatic event, or winging can also develop more slowly due to repetitive heavy lifting or overhead movements. The common pathway to injury seems to be the physical compression of the nerve by the fibers of the middle scalene muscle, which has been implicated in similar compression neuropathy of the dorsal scapular nerve [3,4].

Decompression and microneurolysis of the long thoracic nerve has be shown to be an effective treatment solution for the problem of winging scapula caused by serratus anterior paralysis in cases where nerve injury exists[4]. It has good success functionally both in our unpublished results and in others' experience[3,4]. It is more physiologic and carries significantly less morbidity than other surgical interventions for winging such as pectoralis to scapula tendon transfers or scapulothoracic fusion. An interesting phenomenon seen with some of our patients is the almost immediate improvement in shoulder flexion and abduction following decompression, microneurolysis and tetanic stimulation of the long thoracic nerve. We evaluated all cases with video follow-up for early improvement in active shoulder abduction, and report on 13 instances where serratus anterior function was significantly improved within 24 hours of surgery for long thoracic nerve neuropathy.

## Methods

### Patients

Microneurolysis of the long thoracic nerve was performed 107 times over the past 7 years on 98 patients presenting with winging scapula: 9 patients had bilateral winging and documented long thoracic nerve injury. All cases with documented follow-up within 24 hours of surgery were evaluated for angle of active shoulder abduction. Here we report data on abduction 24 hours after 13 of these surgeries in which an increase in abduction angle of 10% or greater in one day was recorded.

5 patients were men (1 with bilateral winging) and 7 were women, for a total of 13 operations. 1 operation was on the left side and 12 on the right side. The average age of injury was 2.9 years at the time of surgery, ranging from 2 months to 12 years. The onset is approximate as in some cases the symptoms were not noticed immediately following a traumatic injury or other specific inciting event. The average patient age was 37 years, ranging from 15 to 62 years old.

### Surgery

Patients were placed in the lawn-chair position with a shoulder roll. A skin incision was created superior and parallel to the clavicle. Dissection was carried through the platysma muscle while protecting the underlying supraclavicular nerves. Retraction of the omohyoid muscle allowed access to the scalene fat pad, and elevation of the fat pad revealed the upper brachial plexus.

Exploration of the upper trunk and its trifurcation into the anterior and posterior divisions and the suprascapular nerve typically revealed epineurial scarring. The epineurium was released sharply with microsurgical instruments and technique under high magnification.

The long thoracic nerve, lateral and posterior to the upper trunk, was identified within the substance of the middle scalene muscle. Partial resection of the middle scalene was performed to reveal the long thoracic nerve and remove the circumferential muscle fibers. This partial resection of the middle scalene to decompress the LTN released only the most superficial fibers compressing the upper trunk, typically 15%–20% of the thickness of the muscle.

A demarcated area of compression was typically apparent toward the point of exit of the long thoracic nerve from the middle scalene muscle, mirroring the experience of others[4]. The site of compression exhibited narrowing and surface neovascularization of the epineurium. External and internal neurolysis of the isolated nerve were performed with microsurgical instruments and the operating microscope because of the nerve's small size (2–3 mm in diameter) and to reduce surgical scar formation. It should be noted that the long thoracic nerve is multifascicular at this level and internal neurolysis is required to achieve the goals of surgery.

The platysma and two skin layers were reconstructed during closure with no drains. Active range of motion of the shoulder and neck was part of the immediate postoperative management, with a goal of full range of motion at or beyond preoperative levels by the third day after surgery.

### Intraoperative monitoring

Nerve conduction was monitored before and after neurolysis. After induction of anesthesia but prior to surgery commencing, needle electrodes were placed within the serratus anterior muscle and resting muscle action potentials were monitored continuously during the operation. Before and after microneurolysis, muscle action potentials were recorded in response to direct electrical stimulation of the long thoracic nerve once it had been identified and exposed. Tetanic stimulation at 47 Hz was also administered intraoperatively, after performing decompression and neurolysis.

Intraoperative muscle action potential testing of the serratus anterior muscle was performed on all patients. The threshold response for obtaining a signal was measured at 2 intervals: (1) after exposure of the long thoracic nerve and prior to decompression and microneurolysis (2) after decompression, microneurolysis and tetanic stimulation of the nerve. Intensities of stimulation ranged from 0.6 to 16.5 mA (most commonly 2.0–2.6 mA) for 0.5 ms. In all cases there was a decrease in the threshold stimulation current required for equivalent measured muscle action potential amplitudes following microneurolysis and decompression.

### Functional evaluation

The angle of humeral elevation on the day before and the day after surgical neurolysis of the long thoracic nerve was captured on video. Stills were taken from video of the patients performing abduction, and the angle between a line parallel to the medial line and the humerus was measured (0° being relaxed at the side and 180° being fully abducted above the head). In most cases the error on the angle measurement was determined to be 4°. In a few cases the error was larger because the patient's attempts at abduction to the maximum degree involved a certain degree of flexion anteriorly, which causes a small overestimation of the angle of abduction. Overestimation was more often a problem in the preoperative measurement angle and gives a minimum determination of the improvement in those cases.

Measurements were made by the same investigator, independent from the surgeon and clinical staff in all cases. Preoperative and postoperative angles were compared using a paired, 2-tailed t-test performed in Microsoft Excel. Averages are given with one standard deviation.

## Results

**Figure 1:**
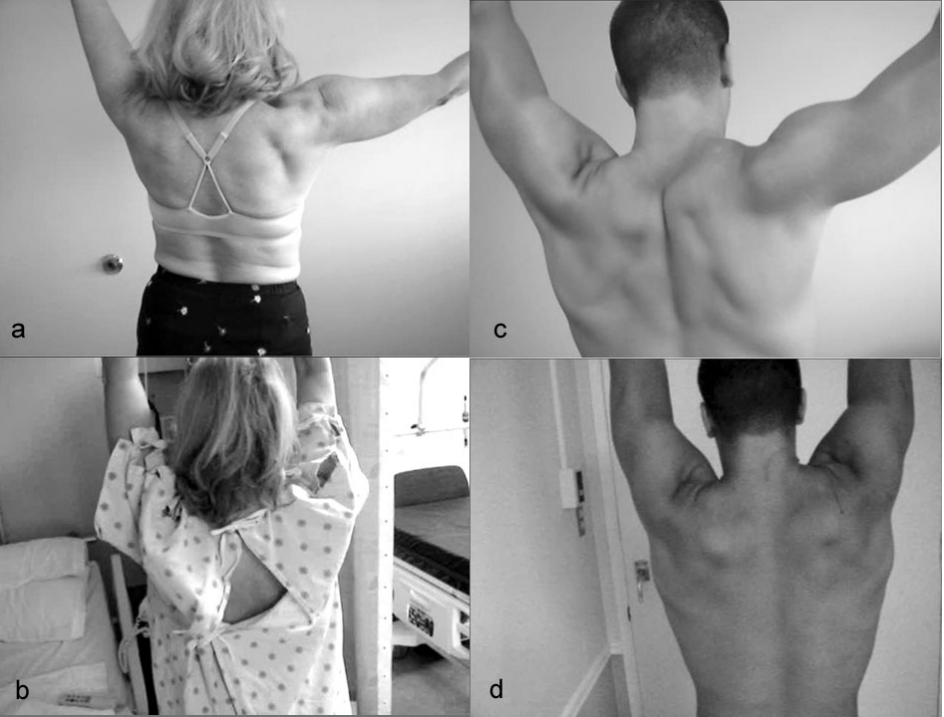
**Abduction improvements one day post surgery**. Video stills showing abduction improvement for two patients in this series. **a, c) **Preoperative maximal humeral elevation of 103° and 134° as seen the day before neurolysis. These patients had been experiencing winging for 4.5 and 1.5 years, respectively. **b, d) **Postoperative abduction of 176° and 149° documented on the day after surgery.

The effect of neurolysis of the long thoracic nerve was studied in 13 surgeries on 12 patients suffering from winging scapula and significantly reduced abduction ability. The ability to abduct the arm on the affected side is reported in Table 1. The average preoperative angle of abduction was 105 ± 30°, and the average postoperative angle of abduction was 164 ± 13°, giving an average improvement of 59 ± 35° within 24 hours (p < 0.00005). All patients were able to abduct their arm to over 145° after neurolysis, whereas before none were able to abduct over 140°. There was no correlation of improvement with the number of years of scapular winging (r < .07). Relative improvement ranged from 10% to 250% above the preoperative angle, with the larger gains seen in patients with the poorest preoperative abduction. Two representative patients with 71% and 11% improvement are shown in Figure 1. Scpular winging was also reduced, as observed clinically, with improved movement of the scapula on the thoracic cage.

**Table 1:** Abduction angle of the affected arm one day prior to and one day following neurolysis of the long thoracic nerve.

Patient	side	sex	Age (yrs)	Age of Injury (yrs)	Overhead Angle Pre-surgery	Overhead Angle Post-surgery	Overhead Angle Increase
1*	l	m	23.3	3.0	47° ± 4	163° ± 8	116° ± 9
2	r	f	54.1	0.6	63° ± 8	153° ± 8	90° ± 11
3	r	f	37.2	1.5	80° ± 8	166° ± 8	86° ± 11
4	r	f	46.6	4.0	81° ± 4	170° ± 4	89° ± 6
5	r	f	15.5	0.8	90° ± 8	180° ± 4	90° ± 9
6	r	f	52.0	4.5	103° ± 4	176° ± 4	73° ± 6
7	r	m	24.2	0.2	120° ± 8	180° ± 4	60° ± 9
8	r	m	22.0	2.0	124° ± 4	147° ± 4	23° ± 6
9	r	m	51.1	12	125° ± 4	170° ± 4	45° ± 6
10*	r	m	23.4	3.0	129° ± 4	146° ± 8	17° ± 9
11	r	f	62.4	4.0	131° ± 4	180° ± 4	49° ± 6
12	r	m	17.6	1.5	134° ± 4	149° ± 4	15° ± 6
13	r	f	51.8	0.4	138° ± 8	152° ± 8	14° ± 11

*Patients 1 and 10 are the same person, who suffered from bilateral winging.

**Figure 2:**
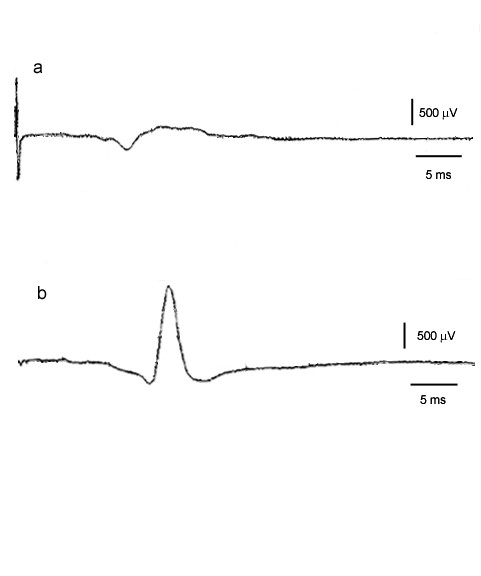
**Intraoperative electrical monitoring**. Intraoperative electrical monitoring of the long thoracic nerve for one, representative patient in this group. **a) **Response of the serratus anterior to a 3.0 mA stimulation before neurolysis. **b) **Response to the same stimulation after neurolysis and tetanic stimulation.

The improvement in abduction is associated with an intraoperative improvement in nerve conduction and muscle contraction. Intraoperative monitoring of muscle action potentials showed definitively improved response of the serratus anterior to electrical stimulation after decompression, microneurolysis and electrical stimulation of the long thoracic nerve. The muscle response increased in amplitude and could also be provoked with a smaller electrical current than before neurolysis. A representative neuromonitoring trace is shown in Figure 2, showing pre-neurolysis response to a 3.0 mA stimulation, and the increased response after neurolysis and tetanic stimulation. This type of result has previously been subjectively reported as a good/improved contraction of the serratus anterior by Disa and coworkers[4].

These 13 operations represents 12% of 107 operations to release and neurolyse the long thoracic nerve performed by one of the authors (RKN). This constitutes a noteworthy proportion of patients who experience an easily measured improvement within the first day. An additional number of patients also experienced rapid improvement following surgery which was not formally quantified by video analysis or resulted in less than 10% change in abduction angle. These patients were not included in this study. The majority of all patients experience improvement within 3 months. The improved overhead movement was retained in all patients at last followup (average 2.3 years, ranging from 8 months to 7 years). Further improvements can be experienced over time with continued physical therapy and electrical stimulation.

## Discussion

Rapid improvement in functional parameters after neurolysis has been described in humans and experimental animals[7,8], including anecdotal case reports involving the long thoracic nerve[4], and ulnar nerve[9]. It is difficult to measure the outcome of surgical procedures when relying on the patients' subjective reports or on postoperative electrical testing, which does not always correlate with function[10,11]. The field remains skeptical of reports even from experienced surgeons[4]. In many cases of long thoracic nerve injury, it is possible to quantitatively measure improvement by following changes in the angle at which the arm can be abducted. A small change in muscle contraction (25% of maximum voluntary contraction) can lead to an easily-measured change in abduction angle (90°)[2]. We used this measurement as a convenient indicator of early recovery 24 hours post-microneurolysis. In this retrospective study, 12 patients undergoing a total of 13 neurolysis surgeries of the long thoracic nerve increased their angle of abduction by an average of 59° within one day of the procedure (Table 1).

In complete axonal and demyelinating injuries, the time to recovery is several weeks to months. In the current series, therefore, the injury is not axonal and apparently not demyelinating although preoperative electromyograms were interpreted to suggest some degree of demyelination in most cases. The nerve must, therefore, be intact, and the muscle must be able to receive signal, otherwise the immediate ability to abduct the arm to a higher degree would not be possible. It is possible that a mixed injury pattern is present with sufficient numbers of intact axons present to produce rapid recovery after surgery. Nevertheless, rapid return of movement following microneurolysis is an interesting and real phenomenon that deserves further consideration and explanation.

When similar observations of early recovery are made, they may be dismissed as coincidental, spontaneous recovery. By directly measuring the nerve conduction before and after surgical procedures, we determined that the nerve was not able to function properly until after the decompression, neurolysis and tetanic stimulation. The persistence of symptoms in patients experiencing scapular winging for longer than 2 years also indicates that recovery was probably not due to the completion of nerve regeneration coinciding with surgical intervention.

It has been observed in several clinical situations that anatomically intact peripheral nerves may be unable to activate target muscles. In the ulnar nerve, for example, nerve conduction is commonly reported to be impeded only at a site of compression caused by fibrosis[9,12]. Since some lesions in continuity are able to be reversed even after an extended period of paralysis, the nerve-muscle connection is probably maintained by chemical factors. Monitoring the electrical response of muscles, McComas reports evidence for variations in the excitability at the neuromuscular junction that appeared to be changes in innervation, but could not have been [13-15]. He concluded that the cause was more likely to be a channel defect in the axolemma, similar to the proposed cause of double crush syndrome[16,17].

It is convenient for diagnosis and prognosis purposes to categorize nerve injuries. The mildest category of injury, neurapraxia, comprises a continuum of increasingly severe injuries to an intact nerve that do not result in Wallerian degeneration of the axon. The observed rapid recovery of muscle function needs to be accounted for more specifically, since recovery from commonly-reported neurapraxic injuries involves remyelination, which takes weeks. It is, therefore, useful to further describe categories of injury at the very mildest end of the neurapraxia spectrum, whatever the underlying cause may be[15,18].

**Figure 3:**
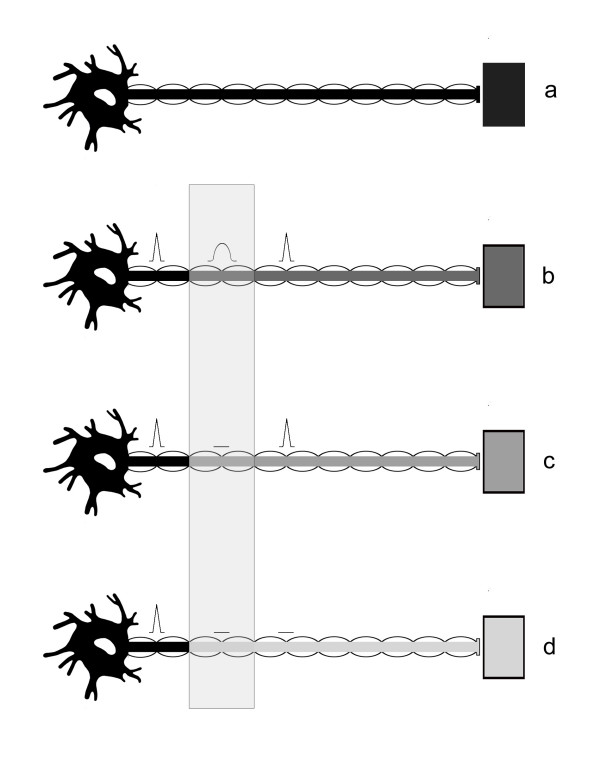
**Possible types of neuropathic disorder**. **a) Normal motoneuron. b) **The **sub-threshold **injury manifests as a slowing of electrical conduction over the site of compressive lesion and a reduction in axonal transport. Both factors could result in reduced muscle response. **c) **In the **local neurapraxic **lesion, conduction is eliminated only over the affected portion of the nerve, and axonal transport is still able to prevent denervation of the muscle. Muscle response is eliminated, since the impulse is blocked at the site of compression. **d) **The **extended neurapraxic **lesion describes a case where the axonal transport is even further decreased, and distal nerve portions are no longer able to carry electrical impulses. Demyelination may or may not occur. There is, however, just enough material transported to maintain a connection to the muscle and prevent nerve degeneration. (adapted from McComas *et al*., 1974 [15]).

McComas describes several possible types of nerve conduction impairment, summarized in Figure 3, that could be due to slowed axonal transport at the site of compression[15]. It is not necessary to specify that all axons innervating the paralyzed muscle are affected to the same extent, and a spectrum of injury severity, with a corresponding spectrum of recovery, is expected to exist within a single nerve. The sub-threshold (Figure 3b) and local (Figure 3c) neurapraxic injuries could be rapidly-reversed once the compression is relieved by neurolysis. If this is the case for a significant proportion of axons in the affected nerve, rapid recovery of muscle function is expected.

A channel defect could easily result in low local concentrations of factors at the motor end plate due to slowed delivery from the nucleus, as depicted in Figure 3. Impaired transport or flow of end plate molecular precursors may result in a situation where the synapse is effectively silent because it is unable to release enough acetylcholine to stimulate muscle contraction. The localization of several neural molecules and cell structures that are affected by nerve compression has been accomplished. Protein distribution, including tubulin, is affected by compression injury to the nerve [18-20]. Molecular transport is inhibited by pressure, and leads to a buildup of proteins at both the proximal and distal sides of the compression, and swelling is observed proximal to the constriction[21]. Both magnitude of the pressure and duration of compression affect severity of symptoms and speed of recovery in experimental models.

There are a large number of proteins and cell components which are necessary for coordinated acetylcholine release at the neuromuscular junction: signaling proteins, vesicle recycling proteins, ATP production and delivery components (including mitochondria). Many, but not all, of these are synthesized exclusively at the soma. The decreased local concentration of any of these factors at the end plate could potentially create a nerve that is unable to stimulate muscle contraction, but is otherwise intact and healthy. Removing the source of the constriction in these cases could allow the nerve to quickly resume muscle stimulation. In the case of this set of surgeries, a rapid recovery of nerve impulse delivery to the serratus anterior is observed once the constriction of the long thoracic nerve is surgically alleviated.

## Conclusion

This series of patients demonstrates that even several years after onset of scapular winging, the long thoracic nerve and serratus anterior muscle can retain the ability recover function within a brief period of time. Although the exact physiological reasons for the phenomenon are unknown, the observed rapid recovery is real, and good background research exists to describe potential mechanisms for this phenomenon. Further inquiry into this area of neurophysiology is needed.

## Competing interests

The author(s) declare that they have no competing interests.

## Authors' contributions

RKN conceived of the study and performed all surgeries. SEM analyzed patient movements from video and drafted the manuscript. All authors read and approved the final manuscript.
